# Effectiveness of screened ceilings over the current best practice in reducing malaria prevalence in western Kenya: a cluster randomized-controlled trial

**DOI:** 10.1017/S0031182022000415

**Published:** 2022-06

**Authors:** Noboru Minakawa, Hitoshi Kawada, James O. Kongere, George O. Sonye, Peter A. Lutiali, Beatrice Awuor, Rie Isozumi, Kyoko Futami

**Affiliations:** 1 Institute of Tropical Medicine (NEKKEN), Nagasaki University, Nagasaki 852-8523, Japan; 2 Kenya Medical Research Institute, Nairobi, Kenya;; 3 Center for Research in Tropical Medicine and Community Development (CRTMCD), Nairobi, Kenya; 4 Ability to Solve by Knowledge Project, Mbita, Kenya

**Keywords:** Anopheline, ceiling, Kenya, malaria, net, RCT

## Abstract

Increases in bed net coverage and antimalarial treatment have reduced the risk of malaria in sub-Saharan Africa. However, the pace of reduction has slowed, and new tools are needed to reverse this trend. We evaluated houses screened with insecticide-treated ceiling nets using a cluster randomized-controlled trial in western Kenya. The primary endpoints were *Plasmodium falciparum* PCR-positive prevalence (PCR*Pf*PR) of children from 7 months to 10 years old and anopheline density. Ceiling nets and bed nets were provided to 1073 houses, and 1162 houses were provided with bed nets only. The treatment and control arms each had four clusters. We conducted three epidemiological and entomological post-intervention surveys over the course of a year and a half. Each epidemiological survey targeted 150 children per cluster, and entomological surveys targeted 25 houses. When the three surveys were combined, the median PCR*Pf*PRs were 23% (IQR 8%) in the intervention arm and 42% (IQR 12%) in the control arm. The adjusted risk ratio (RR) was 0.53 [95% confidence interval (CI) 0.41–0.71; *P* = 0.029]. The median anopheline densities were 0.4 (IQR 0.4) and 2.0 (IQR 1.4), respectively. The adjusted RR was 0.41 (95% CI 0.29–0.90; *P* = 0.029). The present study indicates additional protection from insecticide-screened ceilings over the current best practice.

## Graphical abstract

**Figure:**
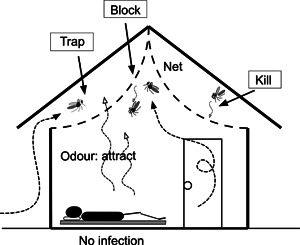

## Introduction

Insecticide-treated bed nets (hereafter termed bed nets) are the most efficient tool for preventing malaria parasite infection in sub-Saharan Africa (ter Kuile *et al*., 2003; Eisele *et al*., 2010; Pryce *et al*., 2018). According to the World Malaria Report 2020 by the WHO (World Health Organization, 2020), half of the people at risk of malaria in sub-Saharan Africa were sleeping under bed nets in 2019, and households with at least one bed net for every two people increased to 36%. With an increase of bed net coverage, the infection prevalence in endemic Africa halved between 2000 and 2015, and the clinical cases fell by 40% (Flaxman *et al*., 2010; Bhatt *et al*., 2015). Although bed nets were continuously delivered to high-risk areas, the rate of reduction slowed considerably after 2014 (Talapko *et al*., 2019; World Health Organization, 2020).

In Africa, children often share one net with more than two persons (Iwashita *et al*., 2010; Minakawa *et al*., 2015; Tamari *et al*., 2019). Even when the number of bed nets is sufficient to cover all family members, their sleeping spaces are often limited for the hanging of nets (Iwashita *et al*., 2010). Under crowded conditions the risk of infection may increase because children touch the net, and extremities extend or the whole body may roll outside the net, especially when sleeping on the floor (Minakawa *et al*., 2015). While babies usually sleep with their mothers on beds, older children may sleep on the floor (Iwashita *et al*., 2010). There, it becomes difficult for the children to properly hang bed nets from ceiling beams and remove them in the morning for day-time activities. Consequently, the children may tie nets to nearby furniture, which can cause the net bottom to spread improperly. A large family may own multiple small buildings or huts, and the practice becomes common in a dwelling without adults. Unless the situation changes, it will be difficult to reach the goals for malaria elimination set by the Global Technical Strategy for Malaria 2016–2030 (Patouillard *et al*., 2017).

Changes in house design may supplement vector control and provide additional protection for persons who cannot effectively use bed nets. Anecdotal reports from the late 19th and early 20th centuries suggested that screening houses reduced the number of mosquitoes entering the house, and as a consequence decreased the prevalence of malaria cases (Lindsay *et al*., 2002). Improved housing with framed glass windows and screens or modern housing may have contributed to the elimination of malaria in North America and Europe (de Zulueta, 1994; Tusting *et al*., 2015). Two recent cluster-randomized control trials (cRCTs) suggest the potential of house screening for malaria control in Africa (Kirby *et al*., 2009; Getawen *et al*., 2018). One study in Ethiopia demonstrated that the rate of malaria was significantly lower [rate ratio 0.38, 95% confidence interval (CI) 0.18–0.82] in houses with screened windows and doors (Getawen *et al*., 2018), but a review considered that the effect had low certainty (Furnival-Adams *et al*., 2020). In The Gambia, the risk ratio (RR) of screening houses (including ceilings) was lower, but the reduction was not statistically significant (RR 0.84, 95% CI 0.60–1.17) despite the significant reduction of anaemia (RR 0.61, 95% CI 0.42–0.89) (Kirby *et al*., 2009; Furnival-Adams *et al*., 2020). Since the previous RCTs did not produce clear results, additional studies are needed to explore the potential of house screening (Gimnig and Slutsker, 2009; Furnival-Adams *et al*., 2020).

**Fig. 1.**
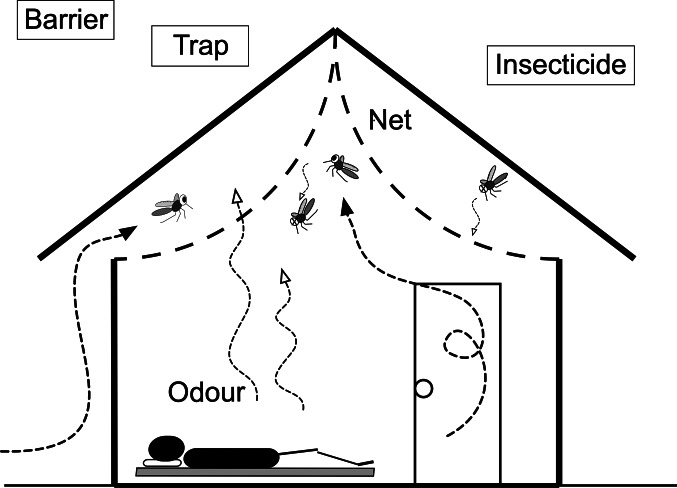
Ceiling net. Mosquitoes that enter from the eave opening are trapped in the space between the ceiling net and roof. Olyset^®^Net, which is treated with permethrin, was used for the ceiling net material. The net may also kill mosquitoes that enter the room from the door and windows.

We evaluated screened ceilings (hereafter termed ceiling nets) within houses using a cRCT in western Kenya. Our study took three approaches which were not practiced by previous studies (Kirby *et al*., 2009; Getawen *et al*., 2018). Firstly, we focused on ceiling nets and did not consider screen windows and doors, because The Gambia study had comparable results between houses fully screened and having ceiling nets only. Since eaves are the major entry route for anophelines (Njie *et al*., 2009), ceiling nets would be more efficient and cost-effective for reducing mosquitoes than full screening. Lindsay *et al*. suggested that ceiling nets may act as decoy traps (Lindsay *et al*., 2003), because most traditional houses in sub-Saharan Africa do not have ceilings but have open eaves, and host odours from the room pass through the nets and may attract mosquitoes from open eaves into the space between the net and roof (Fig. 1). Secondly, since the previous studies did not use an insecticide-treated fabric (Kirby *et al*., 2009; Getawen *et al*., 2018), we used a fabric which is incorporated with 2% permethrin (Olyset^®^Net, Sumitomo Chemical, Tokyo, Japan) (Tamari *et al*., 2020). A study in Brazil reported that *Anopheles darlingi* females prefer to rest on the ceilings (Roberts *et al*., 1987), and a study in the present study area observed that vectors prefer to rest in the upper part of house (Kawada *et al*., 2021). Therefore, we considered that the insecticide-treated ceiling nets are also effective for mosquitoes entering from windows and doors. Finally, we randomized communities instead of households, to add community-level mass effects (community effects) (Gimnig *et al*., 2003; Hawley *et al*., 2003). The primary endpoints were a polymerase chain reaction (PCR) based assay of *Plasmodium falciparum*-positive prevalence (PCR*Pf*PR) of children from 7 months to 10 years old, and the density of anopheline mosquitoes. Secondary endpoints were haemoglobin (Hb) concentration and bed net use; the latter data were used to determine if installing ceiling nets reduces bed net use (Furnival-Adams *et al*., 2020).

## Materials and methods

### Ceiling net

A ceiling net based on the design of Lindsay *et al*. (2003) was manufactured by Sumitomo Chemical using Olyset^®^Net material. Instead of installing the ceiling net parallel to the floor, we lifted the centre of the net and fixed it using a stapler at the highest point of the triangle roof (Fig. 1). This arched arrangement relieves the feeling of oppression under the ceiling net, in particular for tall persons. Lifting the net also reduces the risk of it being torn by activities in the room. As suggested by Lindsay *et al*. (2003), the eaves were not covered with the net so that mosquitoes can enter and become trapped in the space between the net and the roof. Since anopheline mosquitoes have a tendency to rest on the ceiling (Roberts *et al*., 1987; Kawada *et al*., 2021), those entering the room from the doors and windows will become exposed to the net insecticide. Although an arched design requires more netting material, it increases the area to which mosquitoes are exposed. Kawada *et al*. (2012*a*) describe further details of the ceiling net.

### Study area

**Fig. 2.**
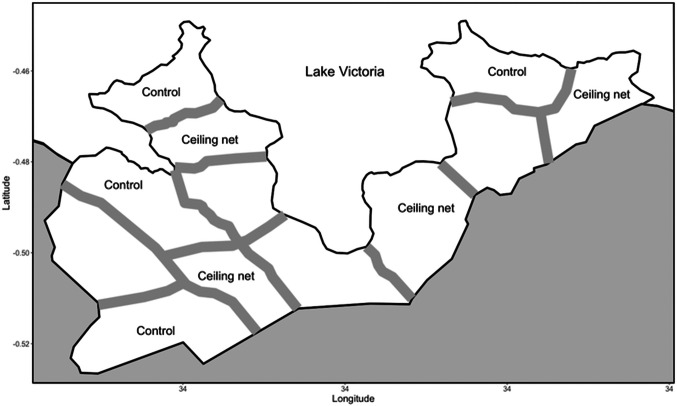
Map showing the boundaries and buffer zones between the intervention and control clusters in the study area.

The study area was Gembe East of Homa Bay County in western Kenya (Fig. 2). The total land area was approximately 46 km^2^, and the coordinates of the geographical mid-point were 0°30′24″ S and 34°20′48″ E. The area was divided into 12 clusters based on 14 communities. The mean area of the clusters was 3.8 km^2^ (s.d. = 0.9). We modified the historical community boundaries to create a ‘fried-egg’ design based on the distribution of children found in the preliminary study (Minakawa *et al*., 2020). Although houses were scarce around the boundaries, buffer zones (300 m) were established to minimize a spill-over effect between clusters based on the flight distance of vectors (Minakawa *et al*., 2002). Most houses are constructed with a stick framework plastered with a mixture of mud and cow dung and a corrugated iron roof. The majority of residents belong to the Luo ethnic group. Dholuo is the main language spoken, although most residents speak English and Kiswahili. The main income sources are fishing, traditional small-scale farming and cattle breeding.

The species of malaria vectors recorded from this study area were *Anopheles arabiensis*, *Anopheles gambiae s.s* and *Anopheles funestus s.s.* When the former two species are grouped as *A. gambiae s.l.*, a previous study in the same area reported that nearly 90% of *A. gambiae s.l.* was *A. arabiensis* (Futami *et al*., 2014). The preliminary study found that 25% of anopheline mosquitoes were *A. funestus s.l.* (Minakawa *et al*., 2020), and a study in an adjacent area reported that nearly all *A. funestus s.l.* individuals collected indoors were *A. funestus s.s.* (Iwashita *et al*., 2014). A small number was also reported of a potential minor vector species, *Anopheles rivulorum*, which belongs to *A. funestus s.l.* (Kawada *et al*., 2012*b*).

A study on insecticide resistance found that over 80% of field-collected *A. gambiae s.s.* had homozygous L1014S mutations associated with knockdown resistance to pyrethroid insecticides, while no single mutation at L1014S was found from *A. funestus s.s.* and *A. arabiensis* mosquitoes collected in this study area (Kawada *et al*., 2011*a*). A survey of field-collected mosquitoes indicated that the *A. funestus s.s.* and *A. arabiensis* populations in the study area have high metabolic resistance to pyrethroid insecticides (Kawada *et al*., 2011*b*). Indoor residual spray was not implemented in the area before and during the present study. Bed nets had been distributed at health facilities, mainly to protect infants and pregnant women, but chemoprophylaxis was uncommon. The common treatment was artemisinin-based combination therapy provided at local health facilities.

Rainfall in the study area follows a bimodal distribution. The long rainy season may start in early March and extend to early June, and the short rainy season may last from October to early December. However, the periods of the rainy seasons vary from year to year.

### Pre-intervention survey (baseline survey)

**Fig. 3.**
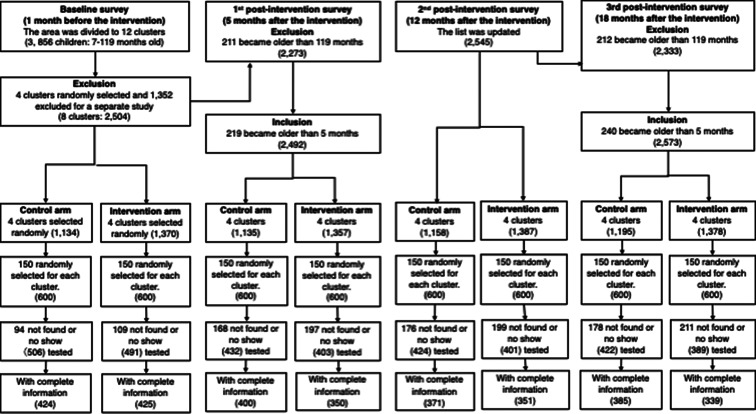
Flow chart and trial profile for epidemiological surveys.

Prior to the epidemiological baseline survey, we held a series of meetings with the local chiefs, village elders and district medical officers in early January 2011, and explained to them the goals of this study. Trained field assistants visited each house and recorded the number of residents, their ages and genders, the number of bed nets and the geographical coordinates with a GPS (Garmin, Olathe, KS, USA). Based on the baseline data we listed children from 7 months to 10 years old, and then selected 150 children from the list for each cluster allocating computer-generated random numbers to all eligible children (Fig. 3).

The preliminary study in 2010 estimated an intraclass correlation coefficient of 0.053 based on a rapid diagnostic test of *P. falciparum*-positive prevalence (RDT*Pf*PR) (Minakawa *et al*., 2020). We expected a 50% reduction of PCR*Pf*PR in the treatment arm. As the RDT*Pf*PR in the study area was 48% in the preliminary study (Minakawa *et al*., 2020), we expected a PCR*Pf*PR of 24% in the treatment arm, assuming the discrepancy between PCR*Pf*PR and RDT*Pf*PR was negligible for sample size calculation. With 80% power and an *α* of 0.05, the estimated cluster size was 116 children. We inflated the sample size to 150 because of anticipated dropouts. The sample size of 150 children was as many as we could adapt, because one cluster had eligible children of fewer than 170. Nevertheless, the number of clusters limits an increase of statistical power (Hemming *et al*., 2011).

Trained field assistants visited the households of the selected children and explained the study to their caretakers and obtained informed written consent. The field assistants informed the caretakers of the primary school and community centre testing locations and dates, and recorded information to assess socioeconomic status (SES). SES for each household was estimated using a composite household material wealth index based on the possession of various consumer goods, house construction, toilet and water access and livestock (Filmer and Pritchett, 2001; Traissac and Martin-Prevel, 2012). A numerical score was assigned to each household using multiple corresponding analysis. The continuous measures were then divided into tertiles to obtain a rough proxy of SES.

Within a few days after consent, we invited the selected children and their caretakers to a testing centre established within each cluster. The axillary temperature of each child was measured, and a finger prick blood sample was taken to conduct RDT (Paracheck-*Pf*, Orchard Biomedical System, Goa, India) for detecting *P. falciparum* infection and to measure Hb concentration (g dL^−1^) using a portable Hb photometer (Hemocue, Angelholm, Sweden). Blood was also drawn into a capillary tube (20 *μ*L) to standardize the blood volume and was preserved on a filter paper. Later, the sampled blood was examined to detect *P. falciparum* using PCR. Artemether-lumefantrine was given to each child who had a positive RDT and body temperature above 37.5°C. Children with Hb concentrations below 11.0 g dL^−1^ were given iron supplementation. Some children whose symptoms did not follow the above criteria were also given the treatment based on the WHO guidelines and diagnosis by a clinician (World Health Organization, 2010). While waiting for an RDT result, caretakers were interviewed on whether their children slept under a bed net the previous night. This is a standard protocol to assess bed net use, and a study in an adjacent area found that the results from interviews for bed net use were similar to those from direct observations in the early morning (Iwashita *et al*., 2010). They were also interviewed on whether children slept on a bed or non-bed sleeping location (Minakawa *et al*., 2015).

For an entomological baseline we used the data from a sentinel surveillance between April 2009 and February 2011. Indoor resting mosquitoes were collected using the pyrethrum spray catch method (PSC) in the morning hours (6:30 to 10:00) from 10 sentinel houses within each cluster. The surveillance sampled mosquitoes every 2 weeks during the period between April 2009 and May 2010, and monthly after May 2010 including the post-intervention period. Details of the sentinel surveillance are described in published studies (Futami *et al*., 2014; Minakawa *et al*., 2020).

### Intervention

In February 2011, after the baseline survey, we selected four clusters for ceiling net intervention and four clusters for control. The remaining four clusters were used for a separate study of bed nets incorporating piperonyl butoxide (Olyset^®^Plus, Sumitomo Chemical). Field assistants visited all listed houses and delivered enough bed nets (Olyset^®^Net, Sumitomo Chemical) in both the control and intervention arms based on the WHO recommendation of at least one bed net for every two persons (Tamari *et al*., 2019). For houses with an odd number of persons we provided extra nets to ensure that all persons had access to a net (e.g. two nets for three persons, and three nets for five persons). In the control arm ceiling nets were added to houses which had structures amenable to installation. The intervention arm received both bed nets and ceiling nets while the control arm received bed nets only.

### Post-intervention survey

We conducted the first post-intervention epidemiological survey in July 2011, after the long rainy season, following the same procedure as the baseline survey. Prior to the survey the same number of children was randomly selected from the list created in January 2011 (Fig. 3). We updated the list of children through another house survey in January 2012, after the short rainy season, and the epidemiological survey was then repeated. The survey was also conducted in July 2012.

To compare entomological data between both arms, we used the monthly data from 80 sentinel houses (10 for each cluster) during the period between March 2011 and May 2012. We also conducted a cross-sectional survey with 25 randomly selected houses in each cluster in May 2011, at the end of the long rainy season. The preliminary study estimated that the number of anophelines per house was 4.3, and the between-cluster coefficient of variance was 0.192 for anopheline mosquitoes (Minakawa *et al*., 2020). The previous study of screened ceilings in experimental hut trials reported an 80% reduction of house entering by *A. gambiae s.l.* (Lindsay *et al*., 2003), and the RCT in The Gambia reported a 47% reduction of *A. gambiae s.l.* (Kirby *et al*., 2009). Therefore, we expected at least 50% reduction from the density of 4.3 per house in the treatment arm. With the sample size of 25 and an *α* of 0.05, the power was 94%. All selected houses were made of mud walls and consisted of one room. Indoor resting female anophelines were sampled using PSC. The cross-sectional survey was repeated with newly selected houses in December 2011 at the end of the short rainy season and June 2012 at the end of the long rainy season. Sampled anophelines were divided into *A. gambiae s.l.* and *A. funestus s.l.* under the microscope, and their numbers were recorded. The sampled houses were selected allocating computer-generated random numbers to all houses listed in each survey.

### Data management

Three independent field teams collected entomological data, epidemiological data and house-related data including survey of bed nets. The data were recorded on paper forms. Two persons converted the data to a digitized form, and the data were independently verified. When discrepancies or missing data were found, the assistants were sent back to the field to confirm or recollect data if possible. All houses, children and bed nets were coded, and the finalized data were stored in a database in Nagasaki University for analyses and security.

### Randomization and blinding

We asked a volunteer to randomize clusters and individuals. Since we did not explain the project to the volunteer, the process was blind. The randomizations were unstratified. The person used a computer program (Stata Statistical Software, Release 11, College Town, TX, USA, StataCorp LP) for the randomizations. The randomization process was explained to the local stakeholders in the community meetings. The persons involved in the epidemiological surveys were not told whether children were from a control group or treatment group. Since ceiling nets were easily recognized once field workers entered the houses, it was impossible to make their activities blind, especially the entomological surveys. However, mosquitoes were collected from each house by a group of 4–5 persons who had been trained, and the activity unlikely induced a bias.

### Statistical analysis

We applied the two-stage procedure for evaluating the effectiveness of ceiling nets on the primary epidemiological endpoint (PCR*Pf*PR) and the secondary endpoint (Hb concentration) comparing the post-intervention data between the two arms based on cluster-level summaries. The two-stage procedure minimizes a loss of statistical power when the number of clusters is small (Bennett *et al*., 2002; Hayes and Moulton, 2017). In the first stage we constructed an individual-level logistic regression model that was adjusted for confounders using R with the package lme4 (Bates *et al*., 2015; R Core Team, 2018). The confounders were age, bed net use, sleeping location, SES and the baseline prevalence data. Using the fitted model, a fitted value was summarized for each cluster. In the second stage we computed a residual from the fitted values and the observed values for each cluster, and we applied Wilcoxon's rank-sum test ranking cluster-level summaries for evaluating the difference between the two groups with the R package coin (Hothorn *et al*., 2008). To estimate a cluster-level effect size and 95% CI, we used bootstrapping (the bias-corrected accelerated bootstrap percentile) with the R package boot (Canty and Ripley, 2016). Bootstrapping is suitable for estimating effect size and CI, because these values do not assume that a null hypothesis is true (Davison and Hinkley, 1997; Hesterberg *et al*., 2005). A Gaussian linear regression model was used for Hb concentration including the same covariates. We evaluated the absolute difference in Hb concentration between the two groups and estimated the effect size and 95% CIs.

Similarly, the effectiveness of ceiling nets on the entomological endpoint was evaluated comparing the post-intervention sentinel data between the two arms based on cluster-level summaries using the two-stage procedure. In the first stage we used an individual-level regression model to obtain a residual of each cluster that was adjusted for pre-intervention baseline data. We first considered a Poisson regression model using R with the package lme4 because of integer values (Bates *et al*., 2015; R Core Team, 2018). A negative binomial model was applied when data were over-dispersed. We also considered houses and sampling dates as potential random factors, because the same houses were sampled repeatedly in the sentinel surveillance. The two-stage procedure was also applied to the cross-sectional entomological data incorporating the pre-intervention sentinel data as a baseline. We analysed data of each of the two mosquito taxonomic groups separately and combined data as ‘anopheline’.

## Results

### Baseline survey

The house survey in early January 2011 recorded 3352 houses and 12 098 residents in the study area. The median number of houses per cluster was 276 (IQR = 75), and the mean number of residents per house was 3.6 (s.d. = 1.8). Ages were confirmed for 11 125 residents, and 3900 (35%) of them were 7 months to 10 years old. As 44 children were later excluded because their houses were within the buffer zones, the target population became 3856. After the random selection of four clusters for each of the intervention and control arms, the number of eligible children became 2504; and the median number of children per cluster was 313 (IQR = 88) in the baseline survey (Fig. 3).

In the baseline epidemiological survey, 203 (17%) children did not show up at the testing centres, and we tested 997 (83%) of 1200 randomly selected children to test for the presence of *P. falciparum* infection (Fig. 3). We excluded incomplete data of 156 children and analysed the remaining data from 849 (71%) children (Table 1). The number of children tested positive was 543, and the prevalence was 64%. The mean Hb concentration was 10.3 (s.d. = 1.9). The number of children who used bed nets the previous night was 472, and the proportion was 56%.

**Table 1.** Individual-level summary statistics of the variables from the epidemiological baseline (pre-intervention) survey and two post-intervention surveys

	Baseline		After 5 months		After 12 months		After 18 months		Overall (without baseline)	
Variable	Control	Treatment	Control	Treatment	Control	Treatment	Control	Treatment	Control	Treatment
Age (s.d.)	5.1 (3.2)	4.5 (3.2)	4.8 (2.5)	4.9 (2.4)	5.1 (2.8)	5.0 (2.6)	5.2 (2.8)	5.2 (2.8)	5.0 (2.7)	5.0 (2.6)
Gender										
Female	230 (54%)	229 (54%)	204 (51%)	177 (51%)	184 (50%)	163 (46%)	198 (51%)	179 (53%)	586 (51%)	519 (50%)
Male	194 (46%)	196 (46%)	196 (49%)	173 (49%)	187 (50%)	188 (54%)	187 (49%)	160 (47%)	570 (49%)	521 (50%)
Net use										
Used	230 (54%)	242 (57%)	360 (90%)	302 (86%)	340 (92%)	300 (85%)	319 (83%)	278 (82%)	1019 (88%)	880 (85%)
Not used	194 (46%)	283 (43%)	40 (10%)	48 (14%)	31 (8%)	51 (15%)	66 (17%)	61 (18%)	137 (12%)	160 (15%)
SES										
Low	101 (24%)	108 (25%)	105 (26%)	122 (35%)	57 (15%)	46 (11%)	57 (15%)	46 (11%)	222 (19%)	378 (36%)
Middle	228 (54%)	219 (52%)	207 (52%)	141 (40%)	226 (61%)	231 (57%)	226 (61%)	231 (57%)	674 (58%)	403 (39%)
High	95 (22%)	98 (23%)	88 (22%)	87 (25%)	88 (24%)	127 (31%)	88 (24%)	127 (31%)	260 (23%)	259 (25%)
Sleeping location										
Bed	140 (33%)	169 (40%)	136 (34%)	126 (36%)	125 (34%)	141 (40%)	132 (34%)	132 (39%)	393 (34%)	399 (38%)
Non-bed	284 (67%)	256 (60%)	264 (66%)	224 (64%)	246 (66%)	210 (60%)	253 (66%)	207 (61%)	763 (66%)	641 (62%)
PCR										
Negative	174 (41%)	132 (31%)	221 (55%)	256 (73%)	198 (53%)	257 (73%)	274 (71%)	283 (83%)	693 (60%)	769 (77%)
Positive	250 (59%)	293 (69%)	179 (45%)	94 (27%)	173 (47%)	94 (27%)	111 (29%)	56 (17%)	463 (40%)	244 (23%)
Hb g dL^−1^ (s.d.)	10.5(1.8)	10.2(2.0)	10.8 (1.7)	11.1 (1.8)	10.7 (1.6)	10.8 (1.8)	11.2 (1.9)	11.2 (1.9)	10.9 (1.8)	11.0 (1.8)
*N*	424	425	400	350	371	351	385	339	1156	1040

**Fig. 4.**
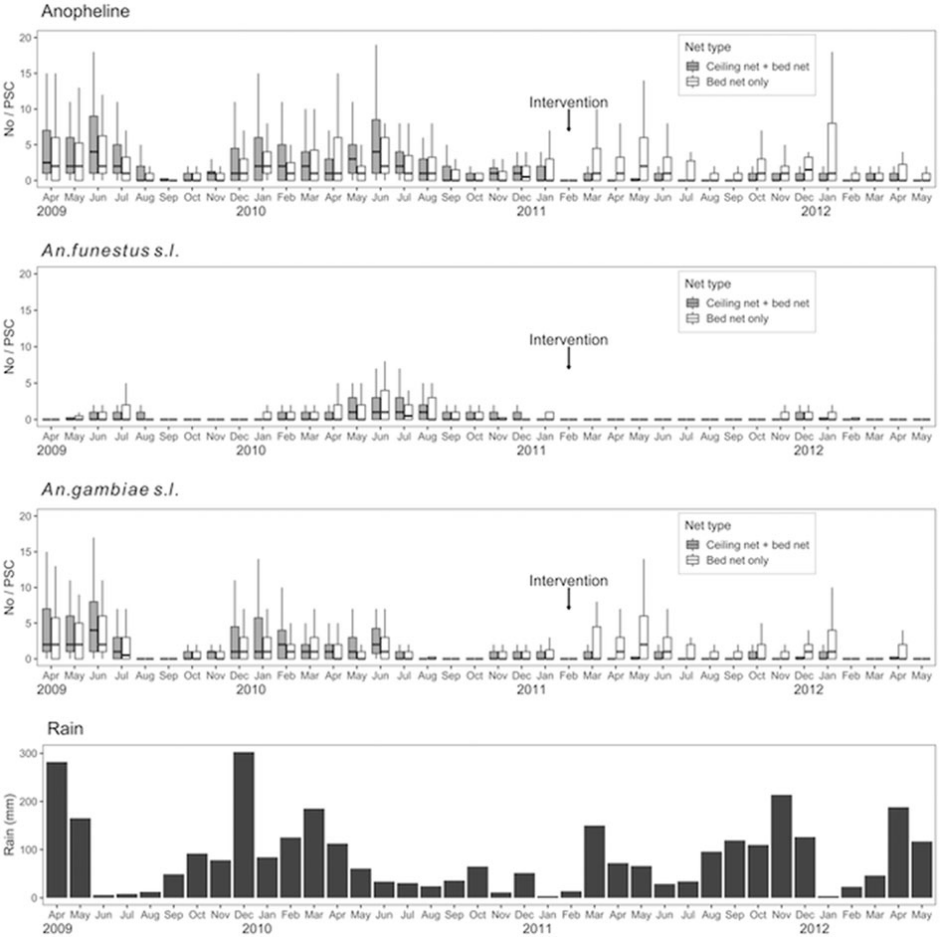
Vector densities from the sentinel house mosquito survey and two post-intervention cross-sectional surveys.

The baseline entomological survey collected 13 620 anopheline mosquitoes from 3200 PSCs at 80 sentinel houses in the randomly selected eight clusters during the period between April 2009 and February 2011. Of them, 3973 (29%) were *A. funestus s.l.*, and 9647 (71%) were *A. gambiae s.l*. The cluster-level median number of anophelines per PSC was 3.8 (IQR = 4.1, *n* = 8), and those of *A. funestus s.l.* and *A. gambiae s.l*. were 0.6 (IQR = 1.8, *n* = 8) and 2.4 (IQR = 2.9, *n* = 8), respectively (Fig. 4).

### Intervention

The number of targeted houses became 2246 after excluding eight houses, because the residents reportedly had migrated to other areas after the baseline survey. The residents of 89 houses were not available during the distribution in the intervention arm, and we could not install ceiling nets and provide them bed nets. As a result, ceiling nets were installed in 1073 of 1162 targeted houses (92%) in the intervention arm and a total of 2260 bed nets were provided. The bed net coverage was 2.1 nets per house and 1.9 persons per net when 89 houses without bed nets were excluded. Including the 89 houses, the figures became 1.9 nets per house and 2.0 persons per net. In the control arm, 2112 bed nets were distributed to 1028 of 1084 targeted houses (95%). The bed net coverage was 2.1 nets per house and 1.7 persons per net when the houses without new bed nets were excluded. When these houses were included, the coverage became 1.9 nets per house and 1.8 persons per net.

### Post-intervention survey

In the first post-intervention epidemiological survey, the individual-level PCR*Pf*PR of the intervention arm was 27% and that of the control arm was 45% (Table 1). The cluster-level analysis revealed that the adjusted PCR*Pf*PR was reduced by 50% (95% CI 14–66%) in the intervention arm when compared with the control arm (Table 2). Despite the range of effect size indicated by 95% CIs, the reduction was not significant with the rank-sum tests (*P* = 0.057). After 12 months of intervention, the individual-level PCR*Pf*PR of the intervention arm was 27%, and that of the control arm was 47% (Table 1). The cluster-level adjusted PCR*Pf*PR was reduced by 41% (95% CI 19–57%) in the intervention arm. The rank-sum tests confirmed that the reduction was statistically significant (*P* = 0.029). The individual-level PCR*Pf*PRs after 18 months were 17 and 28% for the intervention and control arms, respectively. The cluster-level PCR*Pf*PR was reduced by 40% (95% CI 4–67%) in the intervention arm, and the rank-sum test revealed that the reduction was statistically significant (*P* = 0.029). The overall individual-level PCR*Pf*PRs were 23 and 40% in the intervention and control arms, respectively. The cluster-level PCR*Pf*PR was reduced by 47% (95% CI 29–59%) in the intervention arm. The rank-sum test confirmed that the reduction was statistically significant (*P* = 0.029).

**Table 2.** Effects of ceiling nets on PCR*Pf*PR, Hb concentration (g dL^−1^) and bed net use

	After 5 months			After 12 months			After 18 months			Overall		
Variable	Control	Ceiling	*P*	Control	Ceiling	*P*	Control	Ceiling	*P*	Control	Ceiling	*P*
PCR*Pf*PR: median (IQR)	0.52 (0.11)	0.23 (0.09)		0.45 (0.05)	0.28 (0.09)		0.25 (0.16)	0.17 (0.10)		0.42 (0.12)	0.23 (0.08)	
Unadjusted difference	0 (ref)	−0.29 (−0.36, −0.01)	0.057	0 (ref)	−0.17 (−0.30, −0.08)	0.029	0 (ref)	−0.09 (−0.32, 0.14)	0.486	0 (ref)	−0.17 (−0.32, −0.03)	0.057
Adjusted difference	0 (ref)	−0.25 (−0.35, −0.05)	0.057	0 (ref)	−0.18 (−0.26, −0.13)	0.029	0 (ref)	−0.09 (−0.28, 0)	0.057	0 (ref)	−0.19 (−0.26, −0.09)	0.029
Unadjusted risk ratio	1 (ref)	0.47 (0.36, 0.83)	0.057	1 (ref)	0.59 (0.43, 0.81)	0.029	1 (ref)	0.65 (0.24, 1.67)	0.486	1 (ref)	0.57 (0.33, 0.96)	0.057
Adjusted risk ratio	1 (ref)	0.50 (0.34, 0.86)	0.057	1 (ref)	0.61 (0.49, 0.71)	0.029	1 (ref)	0.60 (0.33, 0.96)	0.029	1 (ref)	0.53 (0.41, 0.71)	0.029
Hb median (IQR)	10.7 (0.2)	11.1 (0.4)		10.7 (0.1)	10.8 (0.2)		11.2 (0.4)	11.2 (0.3)		10.9 (0.2)	11.0 (0.2)	
Unadjusted difference	0 (ref)	0.39 (−0.17, 0.77)	0.343	0 (ref)	0.05 (−0.17, 0.41)	0.886	0 (ref)	0.06 (−0.58, 0.46)	0.886	0 (ref)	0.11 (−0.21, 0.40)	0.686
Adjusted difference	0 (ref)	0.25 (−0.19, 0.63)	0.149	0 (ref)	0.12 (−0.05, 0.20)	0.343	0 (ref)	−0.01 (−0.42, 0.28)	1	0 (ref)	0.14 (−0.13, 0.42)	0.343
Bed net use median (IQR)	0.90 (0.04)	0.85 (0.04)		0.92 (0.04)	0.84 (0.05)		0.86 (0.04)	0.81 (0.01)		0.88 (0.02)	0.85 (0.02)	
Unadjusted difference	0 (ref)	−0.04 (−0.10, 0.07)	0.343	0 (ref)	−0.08 (−0.15, 0.01)	0.114	0 (ref)	−0.04 (−0.06, 0.07)	0.343	0 (ref)	−0.04 (−0.08, 0)	0.057
Unadjusted difference	0 (ref)	−0.03 (−0.10, 0.04)	0.486	0 (ref)	−0.07 (−0.13, 0.01)	0.114	0 (ref)	−0.02 (−0.06, 0.09)	0.686	0 (ref)	−0.03 (−0.08, 0)	0.200
Unadjusted net use ratio	1 (ref)	0.95 (0.89, 1.07)	0.343	1 (ref)	0.92 (0.84, 1.01)	0.114	1 (ref)	0.95 (0.93, 1.08)	0.343	1 (ref)	0.96 (0.91, 1.00)	0.057
Adjusted net use ratio	1 (ref)	0.96 (0.89, 1.04)	0.486	1 (ref)	0.92 (0.86, 1.01)	0.114	1 (ref)	0.98 (0.94, 1.13)	0.686	1 (ref)	0.97 (0.92, 1.01)	0.200
*N*	4	4		4	4		4	4		4	4	

The effect sizes and 95% confidential intervals (CIs) were estimated with bootstrapping (the bias-corrected accelerated bootstrap percentile) based on cluster-level median PCR*Pf*PR, Hb concentration and bed net use. The cluster-level differences between two arms were tested with permutation tests. Individual-level regression analyses in the two-stage procedure were adjusted for age, bed net use, sleeping location, SES and baseline prevalence.

The overall cluster-level median Hb concentrations were similar between the intervention and control arms (Table 2). The adjusted difference was 0.11 (95% CI −0.2 to 0.40), and it was not statistically significant (*P* = 0.343).

For bed net use, the overall cluster-level prevalence was over 85% in both arms (Table 2). The net use prevalence was reduced by 3% (95% CI 8–101%) in the intervention arm, but the difference was not statistically significant (*P* = 0.200). Please see a Supplementary file for summaries of the epidemiological endpoints.

During the 15-month post-intervention period, 1865 anopheline mosquitoes were collected from 1200 PSCs in 80 sentinel houses. Of them, 1486 (80%) were *A. gambiae s.l.* and 379 (20%) were *A. funestus s.l.* (Fig. 4). The adjusted RR indicated that the density was reduced by 27% in the intervention arm (95% CI 8–47%) (Table 3). Although the 95% CIs included 1, the difference was not statistically significant with the rank-sum test (*P* = 0.057). The adjusted densities of *A. funestus s.l*. and *A. gambiae s.l.* were reduced by 19% (95% CI 8–133%) and 39% (95% CI 3–50%); however, the reductions were not statistically significant (*P* = 0.200 for *A. funestus s.l*.; *P* = 0.057 for *A. gambiae s.l.*).

**Table 3.** Effects of ceiling nets on median vector densities (number per pyrethrum spray catch)

	Anopheline (total)			*A. funestus s.l.*			*A. gambiae s.l.*		
Variable	Control	Ceiling net	*P*	Control	Ceiling net	*P*	Control	Ceiling net	*P*
Pre-intervention sentinel survey	3.3 (4.6)	3.8 (2.8)		1.5 (2.9)	0.6 (0.4)		1.8 (1.7)	3.3 (2.6)	
Post-intervention sentinel survey	2.5 (1.9)	0.5 (0.3)		0.4 (0.6)	0.1 (0.0)		1.8 (1.0)	0.4 (0.3)	
Unadjusted RR	1 (ref)	0.19 (0.07, 1.21)	0.114	1 (ref)	0.27 (0.09, 0.82)	0.029	1 (ref)	0.20 (0.07, 1.68)	0.114
Adjusted RR	1 (ref)	0.73 (0.53, 0.92)	0.057	1 (ref)	0.81 (0.28, 1.33)	0.200	1 (ref)	0.61 (0.50, 0.97)	0.057
Cross-sectional survey after 3 months	3.1 (4.0)	0.3 (0.7)		0 (0)	0 (0)		3.1 (4.0)	0.3 (0.3)	
Unadjusted RR	1 (ref)	0.08 (0.02, 1.14)	0.114	1 (ref)	NA	1	1 (ref)	0.08 (0.02, 0.51)	0.029
Adjusted RR	1 (ref)	0.31 (0.10, 0.81)	0.029	1 (ref)	NA	0.69	1 (ref)	0.27 (0.10, 0.57)	0.029
Cross-sectional survey after 10 months	1.8 (0.5)	0.3 (0.2)		0.8 (0.5)	0.1 (0.1)		0.7 (0.6)	0.1 (0.1)	
Unadjusted RR	1 (ref)	0.16 (0.08, 0.40)	0.029	1 (ref)	0.16 (0.04, 0.40)	0.029	1 (ref)	0.23 (0.07, 0.50)	0.029
Adjusted RR	1 (ref)	0.70 (0.54, 0.82)	0.029	1 (ref)	0.34 (0.20, 0.61)	0.029	1 (ref)	0.71 (0.48, 0.95)	0.114
Cross-sectional survey after 16 months	1.1 (0.5)	0.5 (0.6)		0.1 (0.1)	0.2 (0.1)		1.0 (0.4)	0.2 (0.4)	
Unadjusted RR	1 (ref)	0.50 (0.14, 1.67)	0.114	1 (ref)	1.6 (0.50, 5.00)	0.26	1 (ref)	0.30 (0.07, 0.91)	0.057
Adjusted RR	1 (ref)	0.72 (0.44, 1.07)	0.2	1 (ref)	1.5 (0.48, 3.82)	0.49	1 (ref)	0.67 (0.40, 1.0)	0.114
Cross-sectional survey: overall	2.0 (1.4)	0.4 (0.4)		0.3 (0.2)	0.1 (0.0)		1.7 (1.4)	0.3 (0.3)	
Unadjusted RR	1 (ref)	0.18 (0.04, 0.69)	0.029	1 (ref)	0.36 (0.16, 0.75)	0.057	1 (ref)	0.13 (0.03, 0.44)	0.029
Adjusted RR	1 (ref)	0.41 (0.29, 0.90)	0.029	1 (ref)	0.71 (0.49, 1.10)	0.114	1 (ref)	0.47 (0.20, 0.67)	0.029
Number of clusters	4	4		4	4		4	4	

The risk ratios (RR) between two arms were tested with a permutation test. The effect size and 95% confidential intervals (95% CIs) were estimated with bootstrapping (the bias-corrected accelerated bootstrap percentile) based on cluster-level median densities (IQR). The individual regression models in the two-stage procedure were adjusted for pre-intervention baseline data. Sampled houses and dates were considered as potential random factors.

Overall, 1130 anophelines were collected in the cross-sectional entomological survey, of which 998 (88%) were *A. gambiae s.l.*, and 132 (12%) *A. funestus s.l*. The cluster-level median densities of anophelines were 0.4 (IQR = 0.4) and 2.0 (IQR = 1.4) for the intervention and control arms, respectively. The adjusted RR indicates that the density in the intervention arm was reduced by 59% (95% CI 10–71%), and the reduction was statistically significant (*P* = 0.029). The densities of *A. gambiae s.l.* were 0.3 (IQR = 0.3) and 1.7 (IQR = 1.4) for the intervention and control arms, respectively; and those of *A. funestus s.l*. were 0.1 (IQR = 0.0) and 0.3 (IQR = 0.2). The density in the intervention arms was reduced by 53% (95% CI 33–80%) for *A. gambiae s.l.*, and by 29% (95% CI 51–110%) for *A. funestus s.l*. The rank-sum tests showed that the reductions were statistically significant for *A. gambiae s.l.* (*P* = 0.029), but it was not significant for *A. funestus s.l*. (*P* = 0.114). Please see Supplementary file 1 for more detailed descriptions for each entomological survey.

## Discussion

The present study demonstrates that the combination of ceiling nets and bed nets is more effective than bed nets alone in reducing *P. falciparum* infection in children in the study area. When compared with the control arm, the overall cluster-level prevalence was reduced by 47% after intervention, and the effect was apparent throughout the post-intervention period. The results indicate an added effectiveness of ceiling nets to bed nets. While ceiling nets physically protect residents, they allow passage of human odours to attract outside mosquitoes through the eaves, to be trapped and then killed by the insecticide in the space between the roof and ceiling net (Lindsay *et al*., 2003). This could produce the positive result on reducing infection risk in the present study, while The Gambia RCT used untreated nets which did not have a clear impact on parasitaemia (Kirby *et al*., 2009). Ceiling nets treated with an insecticide should also be effective for mosquitoes that enter from windows and doors, because anophelines tend to rest on ceilings or in the upper part of houses (Roberts *et al*., 1987; Kawada *et al*., 2021). Moreover, the difference in randomization may explain the discrepancy between the present study and The Gambia RCT (Kirby *et al*., 2009). Household-level randomization may exclude community effects in The Gambia study (Gimnig *et al*., 2003; Hawley *et al*., 2003).

The rank-sum tests produced non-significant results for the differences in densities of all three taxonomic groups (anopheline, *A. gambiae s.l.* and *A. funestus s.l.*) and between both arms in the post-intervention sentinel survey. While the number (*n* = 1200) of PSCs was large, the number (*n* = 1865) of vectors collected might be relatively too small to produce a statistical significance. The relatively small number of vectors was due to inclusion of dry seasons when few vectors were caught. Since we conducted the cross-sectional entomological surveys at the end of rainy seasons, the effects of ceiling nets were clearer especially for *A. gambiae s.l.* and anopheline. When the number of *A. funestus s.l.* was high 10 months after the intervention, the effect of ceiling nets was evident; however, the overall density appears to be insufficient to produce a statistical significance.

Although it is not appropriate to compare the outcomes before and after the intervention because of temporal variations mainly caused by seasonal climate, the prevalence of the intervention arm was reduced by nearly 70% after 16 months of the intervention compared with the pre-intervention, and that of the control arm was also reduced by 50%. Moreover, the density of anophelines remained lower in both arms during the post-intervention period compared with the pre-intervention period. Cumulative effects of ceiling nets and bed nets may partially explain the reduction, even for that of the control arm. Alternatively, the community effect may explain the reduction in the control arm; specifically, it might be due to a spill-over effect by ceiling nets in the adjacent treatment cluster (Gimnig *et al*., 2003; Hawley *et al*., 2003).

While Hb concentrations increased during the post-intervention period in both arms, the statistical tests did not confirm the added effectiveness of ceiling nets on Hb concentrations. In contrast, screened ceilings reduced anaemia in children in The Gambia study (Kirby *et al*., 2009). The discrepancy may be due to co-infection with other diseases such as schistosomiasis which is common in the present study area (Chadeka *et al*., 2019).

Although the bed net use prevalence was slightly less in the intervention arm, the difference was not significant. Despite the addition of ceiling nets, children retained a custom to sleep under bed nets, which is an encouraging sign. Bed net use data reported by residents may bias the result. However, a study in an adjacent area found that the results from interviews for bed net use were similar to those from direct observations in the early morning (Iwashita *et al*., 2010).

### Limitation

The number of clusters was limited to four for each arm, because of the size of the study area. The number of clusters is the minimum requirement to obtain a two-sided *P* value of <0.05 with the rank-sum test based on cluster-level summaries (Hayes and Moulton, 2017). We applied the two-stage procedure adapting the baseline data, and adjusted for covariates such as age and SES in the epidemiological analyses (Bennett *et al*., 2002; Hayes and Moulton, 2017). The purpose of the adjusted analysis is to increase the precision of the cluster-specific estimates. Although the analyses produced a *P* value of <0.05, an unknown factor can still bias the results.

Since parametric tests are not adequate for the small number of clusters, we used the non-parametric tests for null hypothesis testing (Hayes and Moulton, 2017). However, the small number of clusters still produced discrepancies between the *P* values with the rank-sum tests and CIs with bootstrapping (Hesterberg *et al*., 2005).

On the other hand, the small area size allowed us to better control the study; for example, we were able to control the net distribution door to door. With consent we removed nearly all nets already owned and replaced them with new nets to prevent a potential bias from mixing them.

We established a 300 m buffer zone, because a study in an adjacent area reported that over 90% of anophelines were collected in compounds within 300 m of larval habitats. The area sizes and boundary shapes of the clusters also limited the buffer zone to 300 m. Although the buffer zone may also be justified by a mark–release–recapture study in coastal Kenya, which found most mosquitoes in compounds between 200 and 400 m from the release points, the effects of ceiling nets may become clearer with a wider buffer zone. A cRCT of bed nets in western Kenya observed that a malaria-related protection effect and reduction of vector abundance were significant in compounds of control villages lacking bed nets within 600 m of intervention villages (Gimnig *et al*., 2003; Hawley *et al*., 2003).

The species composition of mosquitoes is often spatially heterogeneous. We did not identify collected mosquitoes to the species level; however, a previous study confirmed that nearly 90% of *A. gambiae s.l.* was *A. arabiensis* in the study area (Futami *et al*., 2014). In the adjacent study area, nearly all *A. funestus s.l.* individuals were *A. funestus s.s.* (Iwashita *et al*., 2014). *Anopheles arabiensis* is more zoophilic and exophilic than *A. funestus s.s.* and *A. gambiae s.s.* (Githeko *et al*., 1996). Although the difference in the behaviours could affect the results with the small number of clusters, randomizing the clusters should still reduce the bias.

More than 25% of the selected children were not found or did not show up at the testing centres. There might be several reasons for missing children. They might have migrated to other areas during the short period after the house survey. Parents and caretakers often change children's names in this area, or children have a few names or nicknames, which makes it difficult for field assistants to identify them. Additionally, caretakers might have been too busy to take their children to the testing centres. The missing children would have reduced the statistical power to detect the effect, especially when the numbers of missing children significantly vary among the clusters. At least the effects due to missing children should have been minimized by randomizing the clusters. As the analyses clearly revealed the effectiveness of ceiling nets, to a certain degree adjusting for the confounders such as SES should have taken care of the potential bias related to missing children. However, if the missing children were different in a certain characteristic from the tested children, the epidemiological findings of the present study might not apply to them.

## Conclusion

The present study indicates the effectiveness of ceiling nets in reducing indoor resting vectors and PCR*Pf*PR in children from 7 months to 10 years old. Considering the recent slowed pace of malaria reduction, ceiling nets may become an additional tool for strengthening the current malaria control programmes. However, this new tool needs to be tested further under different geographical locations and settings, and in a larger study area. The cost-effectiveness of ceiling nets also needs to be assessed.

## Data Availability

The data that support the findings of this study are available from the corresponding author (N. M.), upon reasonable request.

## Supplementary material

For supplementary material accompanying this paper visit https://doi.org/10.1017/S0031182022000415. click here to view supplementary material
