# Supplementary material for: Effectiveness of screened ceilings over the current best practice in reducing malaria prevalence in western Kenya: a cluster randomized-controlled trial
Source: Parasitology. 2022 Apr 19;149(7):944–55. doi: 10.1017/S0031182022000415 (PMC10090608; doi:10.1017/S0031182022000415)
Supplement: Supplementary file 1 [file S0031182022000415sup.zip › S0031182022000415sup001.docx]

**Supplementary file 2. Summaries of the epidemiological endpoints for each cluster.**

|  |  |  | Control arm | | |  |  |  |  |  | Treatment arm | | |  |  |
| --- | --- | --- | --- | --- | --- | --- | --- | --- | --- | --- | --- | --- | --- | --- | --- |
|  | Cluster 1 | Cluster 2 | | Cluster 3 | Cluster 4 | | Total |  | Cluster 1 | Cluster 2 | | Cluster 3 | Cluster 4 | | Total |
| PCR Positive (%) |  |  | |  |  | |  |  |  |  | |  |  | |  |
| Baseline | 73 (77%) | 60 (71%) | | 72 (48%) | 45 (48%) | | 250 (59%) |  | 98 (84%) | 56 (52%) | | 88 (76%) | 51 (60%) | | 293 (69%) |
| After 5 months | 49 (56%) | 52 (54%) | | 43 (50%) | 35 (27%) | | 179 (45%) |  | 34 (41%) | 16 (20%) | | 17 (21%) | 27 (26%) | | 94 (27%) |
| After 12 months | 51 (56%) | 40 (45%) | | 34 (41%) | 48 (44%) | | 173 (47%) |  | 29 (32%) | 25 (20%) | | 24 (35%) | 16 (25%) | | 94 (27%) |
| After 18 months | 33 (31%) | 47 (47%) | | 18 (21%) | 13 (14%) | | 111 (29%) |  | 20 (34%) | 9 (8%) | | 13 (19%) | 14 (15%) | | 56 (17%) |
| Overall (without. baseline) | 133 (47%) | 139 (49%) | | 95 (37%) | 96 (29%) | | 463 (40%) |  | 83 (36%) | 50 (15%) | | 54 (25%) | 57 (22%) | | 244 (23%) |
| Hb (SD) |  |  | |  |  | |  |  |  |  | |  |  | |  |
| Baseline | 10.2 (1.8) | 10.4 (2.0) | | 10.7 (1.8) | 10.4 (1.8) | | 10.5 (1.8) |  | 10.1 (2.0) | 10.6 (2.3) | | 10.0 (2.0) | 10.3 (1.9) | | 10.2 (2.0) |
| After 5 months | 10.7 (1.4) | 10.6 (1.8) | | 10.7 (1.9) | 11.0 (1.7) | | 10.8 (1.7) |  | 11.0 (1.6) | 11.4 (1.7) | | 10.6 (1.7) | 11.2 (1.9) | | 11.1 (1.8) |
| After 12 months | 10.4 (1.6) | 10.7 (1.5) | | 10.9 (1.7) | 10.7 (1.6) | | 10.7 (1.6) |  | 10.6 (1.7) | 10.8 (1.9) | | 10.8 (1.7) | 10.7 (1.9) | | 10.8 (1.8) |
| After 18 months | 10.9 (2.0) | 11.1 (1.8) | | 11.3 (1.9) | 11.7 (1.9) | | 11.2 (1.9) |  | 11.3 (1.9) | 11.4 (1.9) | | 10.9 (1.8) | 11.1 (1.9) | | 11.2 (1.9) |
| Overall (without baseline) | 10.7 (1.7) | 10.8 (1.7) | | 11.0 (1.8) | 11.1 (1.7) | | 10.9 (1.8) |  | 10.9 (1.7) | 11.2 (1.9) | | 10.8 (1.7) | 11.1 (1.9) | | 11.0 (1.8) |
| Bed net use (%) |  |  | |  |  | |  |  |  |  | |  |  | |  |
| Baseline | 37 (39%) | 53 (62%) | | 77 (51%) | 63 (67%) | | 230 (54%) |  | 62 (53%) | 62 (58%) | | 72 (62%) | 46 (54%) | | 242 (57%) |
| After 5 months | 79 (91%) | 91 (95%) | | 74 (86%) | 116 (89%) | | 360 (90%) |  | 70 (85%) | 69 (85%) | | 78 (95%) | 85 (81%) | | 302 (86%) |
| After 12 months | 87 (96%) | 77 (88%) | | 74 (90%) | 102 (93%) | | 340 (92%) |  | 78 (85%) | 115 (91%) | | 53 (78%) | 54 (83%) | | 300 (85%) |
| After 18 months | 79 (75%) | 86 (85%) | | 75 (86%) | 79 (87%) | | 319 (83%) |  | 47 (81%) | 99 (84%) | | 55 (81%) | 77 (81%) | | 278 (82%) |
| Overall (without baseline) | 245 (86%) | 254 (89%) | | 223 (87%) | 297 (89%) | | 1019 (88%) |  | 195 (84%) | 283 (87%) | | 186 (85%) | 216 (82%) | | 880 (85%) |
| Number of children |  |  | |  |  | |  |  |  |  | |  |  | |  |
| Baseline | 95 | 85 | | 150 | 94 | | 424 |  | 117 | 107 | | 116 | 85 | | 425 |
| After 5 months | 87 | 96 | | 86 | 131 | | 400 |  | 82 | 81 | | 82 | 105 | | 350 |
| After 12 months | 91 | 88 | | 82 | 110 | | 371 |  | 92 | 126 | | 68 | 65 | | 351 |
| After 18 months | 106 | 101 | | 87 | 91 | | 385 |  | 58 | 118 | | 68 | 95 | | 339 |
| Overall (without. baseline) | 284 | 285 | | 255 | 332 | | 1156 |  | 232 | 325 | | 218 | 265 | | 1040 |
