# Supplementary material for: Effectiveness of screened ceilings over the current best practice in reducing malaria prevalence in western Kenya: a cluster randomized-controlled trial
Source: Parasitology. 2022 Apr 19;149(7):944–55. doi: 10.1017/S0031182022000415 (PMC10090608; doi:10.1017/S0031182022000415)
Supplement: Supplementary file 1 [file S0031182022000415sup.zip › S0031182022000415sup002.docx]

**Supplementary file 1. Detailed descriptions of results from each cross sectional entomological surveys**.

In the cross-sectional entomological survey after three months of intervention, a total of 774 anophelines were collected from 198 houses (PSC was not conducted in two houses because the residents were not present). Of them, 769 (99%) were *A. gambiae* *s.l,* and 5 (1%) were *A. funestus* *s.l*. The adjusted RRs indicate that the reductions in the intervention arm were 69% (95%CI: 19%, 90%) for anopheline and 73% (95%CI: 43%, 90%) for *A. gambiae* *s.l,* (Table 3). The rank sum tests showed that the reduction was statistically significant for anopheles and *A. gambiae* *s.l.*, but it was not significant for *A. funestus s.l*. The number of *A. funestus s.l.* was apparently insufficient to conduct analyses.

After 10 months, the second survey of 199 houses collected 201 anophelines, including 109 (54%) *A. gambiae* *s.l* and 92 (46%) *A. funestus s.l.* The adjusted RRs indicate that the reductions in the intervention arm were 30% (95%CI: 18%, 46%) for anopheline, 66% (95%CI: 39%, 80%) for *A. funestus s.l.* and 29% (95%CI: 5%, 52%) for *A. gambiae* *s.l,* (Table 3). The rank sum tests showed that the reduction was statistically significant for anopheline and *A. funestus s.l.*, but it was not significant for *A. gambiae s.l*.

After 16 months, from 200 houses the survey collected 155 anophelines, including 120 (77%) *A. gambiae* *s.l* and 35 (23%) *A. funestus* *s.l.* The reductions in the intervention arm were 28% (95%CI: -7%. 56%) for anopheline, 150% (95%CI: -282%, 52%) for *A. funestus s.l.* and 33% (95%CI: 0%, 60%) for *A. gambiae* *s.l,* (Table 3). The reductions were not statistically significant.
